# Consumption of cranberry as adjuvant therapy for urinary tract infections in susceptible populations: A systematic review and meta-analysis with trial sequential analysis

**DOI:** 10.1371/journal.pone.0256992

**Published:** 2021-09-02

**Authors:** Jia-yue Xia, Chao Yang, Deng-feng Xu, Hui Xia, Li-gang Yang, Gui-ju Sun

**Affiliations:** 1 Key Laboratory of Environmental Medicine and Engineering of Ministry of Education, School of Public Health, Southeast University, Nanjing, P.R. China; 2 Department of Nutrition and Food Hygiene, School of Public Health, Southeast University, Nanjing, P.R. China; University of Mississippi Medical Center, UNITED STATES

## Abstract

The efficacy of cranberry (Vaccinium spp.) as adjuvant therapy in preventing urinary tract infections (UTIs) remains controversial. This study aims to update and determine cranberry effects as adjuvant therapy on the recurrence rate of UTIs in susceptible groups. According to PRISMA guidelines, we conducted a literature search in Web of Science, PubMed, Embase, Scopus, and the Cochrane Library from their inception dates to June 2021. We included articles with data on the incidence of UTIs in susceptible populations using cranberry-containing products. We then conducted a trial sequential analysis to control the risk of type I and type II errors. This meta-analysis included 23 trials with 3979 participants. We found that cranberry-based products intake can significantly reduce the incidence of UTIs in susceptible populations (risk ratio (RR) = 0.70; 95% confidence interval(CI): 0.59 ~ 0.83; *P*<0.01). We identified a relative risk reduction of 32%, 45% and 51% in women with recurrent UTIs (RR = 0.68; 95% CI: 0.56 ~ 0.81), children (RR = 0.55; 95% CI: 0.31 ~ 0.97) and patients using indwelling catheters (RR = 0.49; 95% CI: 0.33 ~ 0.73). Meanwhile, a relative risk reduction of 35% in people who use cranberry juice compared with those who use cranberry capsule or tablet was observed in the subgroup analysis (RR = 0.65; 95% CI: 0.54 ~ 0.77). The TSA result for the effects of cranberry intake and the decreased risk of UTIs in susceptible groups indicated that the effects were conclusive. In conclusion, our meta-analysis demonstrates that cranberry supplementation significantly reduced the risk of developing UTIs in susceptible populations. Cranberry can be considered as adjuvant therapy for preventing UTIs in susceptible populations. However, given the limitations of the included studies in this meta-analysis, the conclusion should be interpreted with caution.

## Introduction

Urinary tract infections (UTIs) are among the most prevalent bacterial infections in the community, outpatient and inpatient facilities, affecting about 150 million people annually [1]. Generally, UTIs can be categorized into complicated and uncomplicated, or as upper (pyelonephritis) and lower (confined to the bladder). According to U.S. Centers for Disease Control and Prevention, UTIs cause about 13,000 deaths each year [2]. Bacteriuria is a typical feature of UTI, with a high prevalence among young people and an age-related increase in both men and women [1]. Until the age of 60 years and older, the prevalence of bacteriuria is significantly higher in women than in men [3]. Adult women, in particular, are susceptible to UTIs, with nearly 20% to 30% of women with an infection experiencing recurrence [4]. Other populations with a high risk of UTIs include pregnant women, children, elderly patients, participants with indwelling catheters, and patients with neuropathic bladder [5, 6].

Cranberries (*Vaccinium macrocarpon*), originated in New Zealand, are rich in complex phytochemical compositions, such as A-type proanthocyanidins (PACS), anthocyanins, benzoic acid and ursolic acid [7]. *Escherichia coli*, a primary pathogen involved in UTIs, is reported to be prevented from adhering to uroepithelial cells by PACs contained in cranberries in the urinary tract [8, 9]. According to several recent consensus and guidelines on UTI management, the efficacy of cranberry supplementation as an adjuvant therapy has not yet reached a definitive conclusion on preventing and treating UTIs, and the quality of evidence was low. Therefore, it is necessary to assess the effects of cranberry intake on the incidence of UTIs in susceptible groups.

In 2012, Jepson et al. [6] conducted a systematic review of cranberry intake as adjuvant therapy for preventing and treating UTIs, and it was concluded that cranberry products failed to significantly reduce the occurrence of UTIs when compared with placebo or control groups. Additionally, a meta-analysis included 28 clinical studies in 2017 demonstrated that cranberry intake is associated with preventing UTIs, and that supplementing cranberry-based products provides a beneficial effect on reducing the incidence of UTIs [10]. However, few previous studies evaluated the effects of cranberry and UTIs in susceptible populations. Additionally, the efficacy of cranberry intake in preventing UTIs in susceptible individuals is yet not conclusive.

Some new relevant trials with inconsistent conclusions on using cranberry to prevent and treat UTIs have recently been published. The trial sequential analysis (TSA) method was applied to control the inflation of type I error rates. We, therefore, conducted an updated systematic review and meta-analysis with TSA to evaluate the effects of cranberry intake as adjuvant therapy for preventing and treating UTIs in susceptible populations.

## Materials and methods

### Ethics statement

There was no need for ethical approval since all included studies in this systematic review of meta-analysis were officially published in a peer-reviewed journal.

### Literature search strategy

Following Preferred Reporting Items for Systematic Reviews and Meta-Analyses (PRISMA) guidelines, a systematic review and meta-analysis were conducted [11]. Relevant literature was identified through searching systematically in June 2021 in the following five electronic databases: Web of Science, PubMed, Embase, Scopus and Cochrane Library. Search terms included *cranberry*, *Vaccinium macrocarpon*, *Vaccinium microcarpum*, *Vaccinium oxycoccus*, *Vaccinium erythrocarpum* AND Urinary Tract Infection(s), UTI, bacteriuria, pyelonephritis, cystitis, pyuria, dysuria, Escherichia coli, and coli, without language restrictions. Additionally, the reference lists of the eligible articles were further manually retrieved by the reviewers to recognize potentially relevant studies. Data extraction was performed by two reviewers independently (J. Y. X. and C. Y.). Any disagreements were resolved by discussion or, if needed, by consultation with a third review author (X. H.). The details of the search strategy are shown in S1 Table.

### Inclusion and exclusion criteria

Two review authors (J. Y. X. and C. Y.) independently extracted the study data of the eligible articles from the literature search. Original studies were included if they met the following inclusion criteria:(1) study design was limited only to randomized controlled trial; (2) For the intervention used, we only include trials which compared cranberry-containing products to a placebo or non-placebo control group; (3) outcomes which can be calculated or reported as the number of participants experiencing a UTI; (4) The study susceptible populations included participants with recurrent UTIs, elderly men and women, pregnant women, children, participants with indwelling catheter, and participants with neuropathic bladder. The following exclusion criteria were used: (1) the trials whose intervention contained cranberry in combination with another bioactive compound; (2) studies that clearly did not adhere to the aforementioned inclusion criteria; (3) animal studies, case reports, reviews, conference papers, editorials, and studies with insufficient data. Any discrepancy upon inclusion or exclusion of the study was resolved by discussion among the authors (J. Y. X., C. Y. and X. H.).

### Data extraction and methodological assessment

The cumulative incidence of participants with recurrent urinary tract infection(s) was used for pooled risk ratio estimates. Two authors (J. Y. X. and C. Y.) extracted detailed information of study methodology, characteristics of participants, intervention details, and outcomes reported. We assumed the incidence of UTI as the prespecified primary outcome, which was expressed as either incidence or cumulative incidence rate. Trials which compared cranberry-based products to a placebo or non-treatment control were included. Quality assessment of included studies was performed based on the Cochrane Risk of Bias tool [12], and the tool covers seven domains (random sequence generation, allocation concealment, blinding of participants and personnel, blinding of outcome assessment, incomplete outcome data, selective outcome reporting and other bias) and each item was scored as “high risk,” “low risk,” or “unclear” for all selected studies.

### Data synthesis and analysis

Meta-analyses were performed by using Stata SE (15.0) and R (v 4.0.3). Statistical significance was defined as P < 0.05 (2-sided). Data were calculated as risk ratios with 95% confidence interval (95% CI). Heterogeneity within studies was assessed by means of the I^2^ metrics and chi-square statistics, either I^2^ >50% or p value of χ^2^ test <0.10 was considered as statistically significant heterogeneity. We applied a fixed-effect model by Mantel-Haenszel to estimate the summary risk if the heterogeneity was low to moderate (I^2^ < 50%). Otherwise, the random-effects model by DerSimonian and Laird method was used [13]. We conducted subgroup analyses by prespecified covariates, including study design, study analysis, risk of random sequence generation, treatment in control group, population type, age, form of cranberry-containing products, and dose frequency used. A Galbraith plot was constructed to identify potential outlier studies. Funnel plot and egger’s regression were drawn to graphically evaluate publication bias, and a ‘trim and fill’ analysis was used to further observe the stability of results if there is any asymmetry in funnel plot [14].

### Trial Sequential Analysis (TSA)

In order to control the risk of type I and type II errors, a trial sequential analysis method was conducted on the incidence of UTIs. We performed TSA software (version 0.9.5.10 beta) to adjust CIs due to sparse data and repeat testing on the cumulative meta-analysis. If the cumulative Z-curve crosses the TSA monitoring boundary, or enters the Required Information Size line, the result can be concluded that a firm conclusion can be reached and no further studies are needed [15]. TSA was performed at the level of an overall 5% risk of a type I error and with 80% power.

## Results

### Search results and trial characteristics

**Fig 1:**
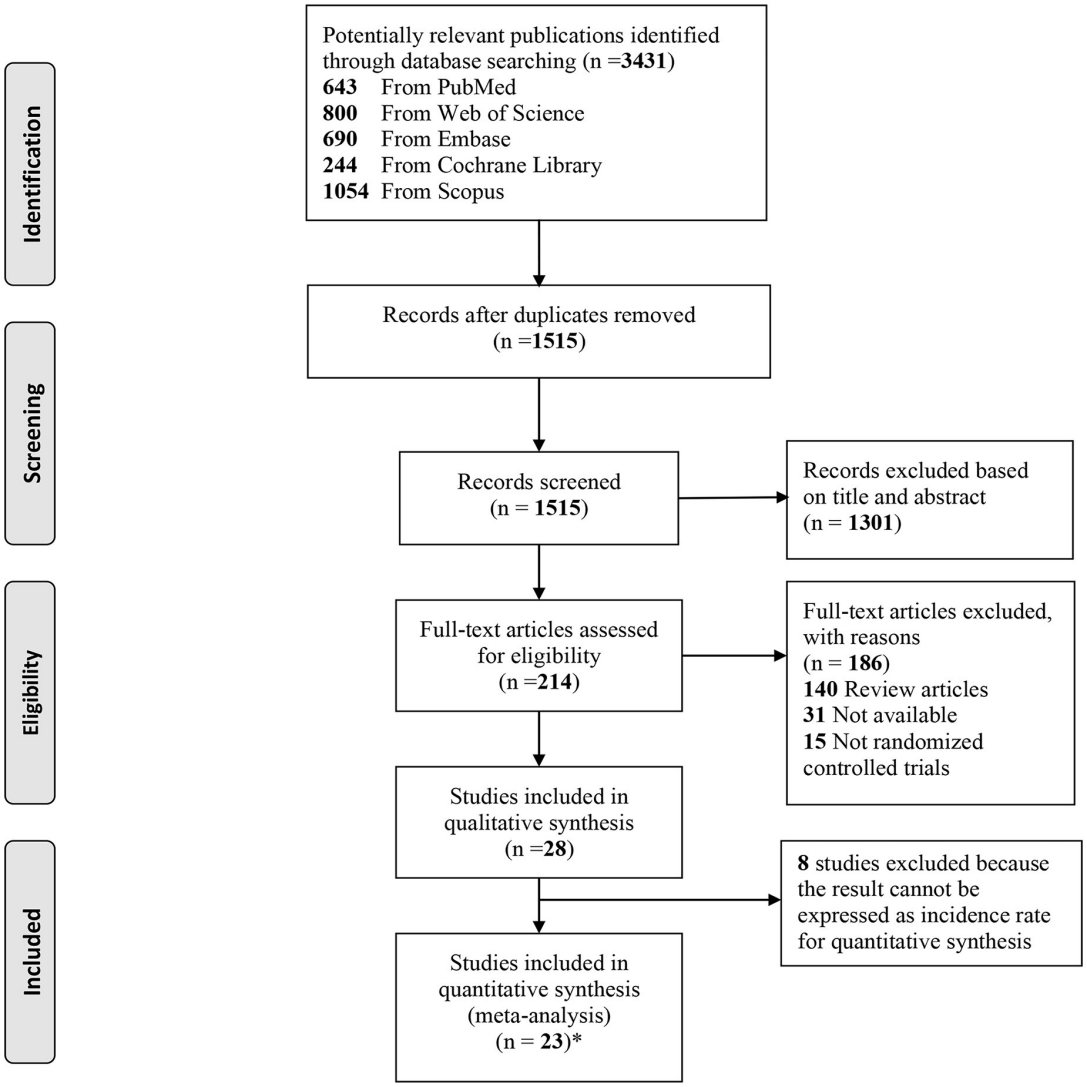
Flow diagram for selected trials. RCT, randomized controlled trial. Asterisk represents that studies by Stothers et al. [16], Caljouw [17] and Wing et al. [18]were each divided into 2 trials.

A flow diagram for included trials is shown in Fig 1. A total of 3431 articles were identified initially with the search strategy from five electronic databases, of which 3217 were removed after reviewing title and abstracts, including 1916 duplications. And then, 214 full-text trials were retrieved for further information; of those, 186 studied were excluded for the following reasons: 140 for review articles, 31 for not available full articles, 15 for non-randomized controlled trials. Finally, eight trials were excluded for not being expressed as incidence rate and three of the 20 included studies were each divided into two trials. The study by Caljouw et al. was divided since it included participants at high and low UTI risk [17]. Stothers used two ways of orally administering cranberry-based products, that is cranberry tablets and cranberry juice [16]. Wing et al. administered two doses of PACs, including a high dose (240 mg PACs per day) and a low dose (80 mg PACs per day) [18]. Therefore, twenty-three trials with sufficient data were eligible for inclusion into the final meta-analysis.

In this systematic review, we identified twenty-eight trials that were eligible for qualitative synthesis. There were 24 parallel-group and 4 crossover group (S2 Table). All of these crossover trials had no washout periods. Nineteen trials were conducted according to the intention-to-treat principle, and 9 trials used per-protocol analysis. Twelve trials did not provide their randomization information, and suffered from a high rate of participants lost to follow-up (0%-48%). In addition, 9 trials had high reporting bias (S3 Table).

There were 4699 subjects included in the qualitative analysis (S4 Table). Of the 28 trials, 16 were performed in North America (United States and Canada), 5 were conducted in Europe (United Kingdom, Finland, and Italy), 2 were performed in the Kingdom of the Netherlands, and 5 separate studies from Japan, Taiwan (China), India, Czech Republic and Turkey. The vast majority of studies followed subjects from Hospital clinic. According to the characteristics of included study subjects, the study population were further categorized into six different subgroups: women with recurrent UTIs, neuropathic bladder, children, pregnant patients, elderly patients and patients with indwelling catheters.

Characteristics of interventions of the included trials are summarized in S5 Table. Cranberry-containing products differed remarkably in cranberry form, manufacturer, daily dosage, PAC content, and dosing frequency. Fifteen trials administered cranberry juice, whereas one trial used both cranberry juice and cranberry tablets, and twelve trials used cranberry capsules. Eleven trials used cranberry-based products from the manufacturer Ocean Spray. Daily cranberry amount ranged from 0.4 to 194.4g and the actual cranberry amount was not reported in the eleven trials. Twenty-three trials used a formula placebo, whereas 5 trials did not used a placebo.

Differences were most notable in the definitions of UTI (S6 Table). Clinical symptoms to define UTI were required in most trials, and the baseline bacteriuria were not excluded in eighteen trials; In addition, the thresholds of bacteriuria ranged from ≥10^3^ to ≥10^5^ CFU/mL. The presence of UTI symptoms was not required in eleven trials. Because there were no reliable biological detection methods to examine the compliance of subjects, most studies use indirect methods for purpose. These included periodic interviews, self-reported questionnaires and recording the number of remaining pills. In most trials, the occurrence of UTI was expressed as incidence or cumulative incidence. There were four trials for which we were unable to calculate and obtain cumulative incidence, and four trials did not provide specific number of events. Finally, eight trials were excluded, and a total of twenty-three trials were further analyzed in quantitative synthesis.

### Quantitative data synthesis

**Fig 2:**
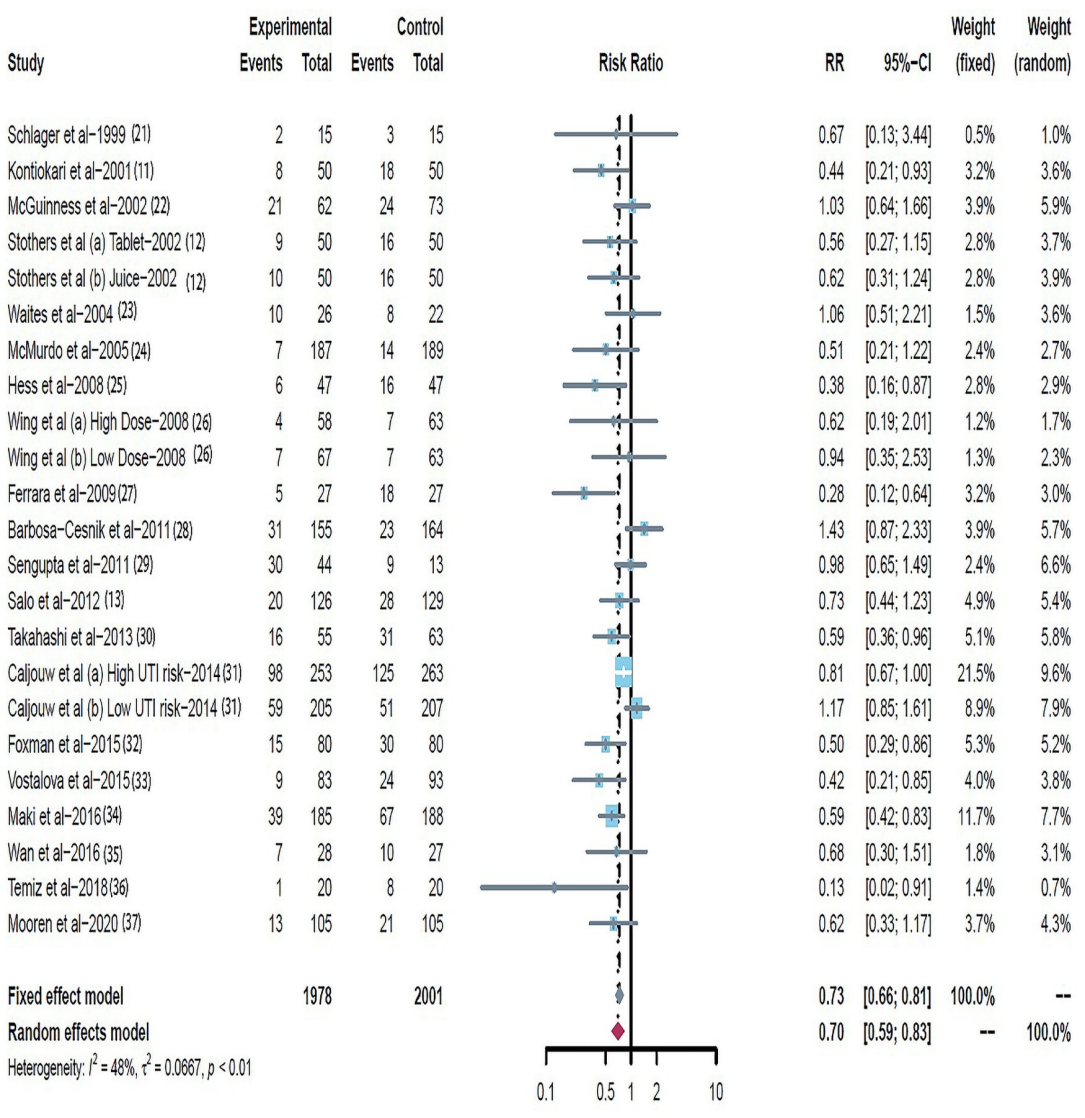
Forest plot: Summary effect of cranberry in prevention of urinary tract infection, expressed as risk ratio (RR). Weights are from random-effect analysis.

**Fig 3:**
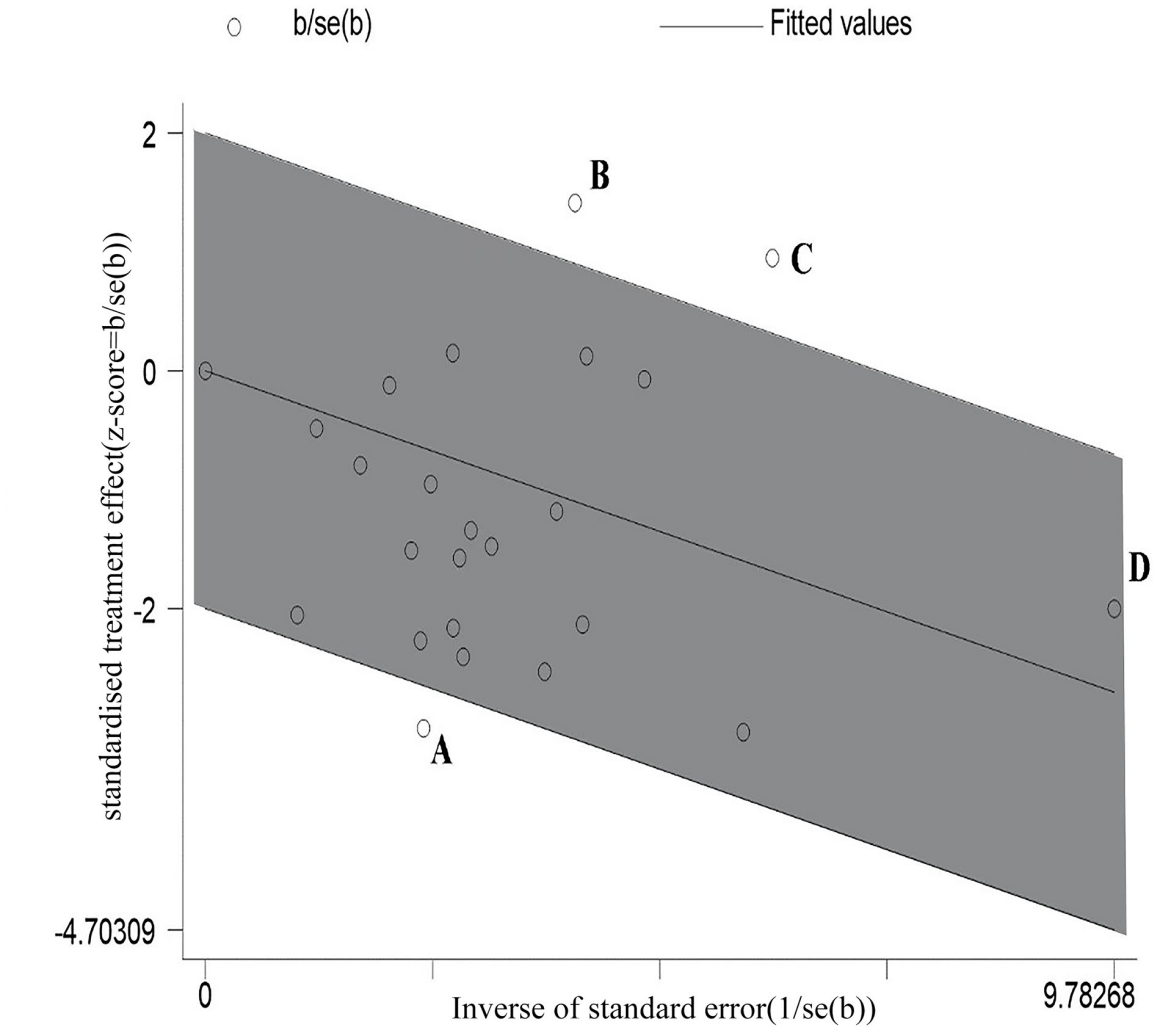
Galbraith plot. There are 3 statistical outliers, A, B and C, which represent the trials by Ferrara et al., Barbosa-Cesnik et al., and Caljouw et al. (b) Low UTI risk, respectively. And there is a statistical extreme point, D, which represent the trial by Caljouw et al. (a) High UTI risk.

**Fig 4:**
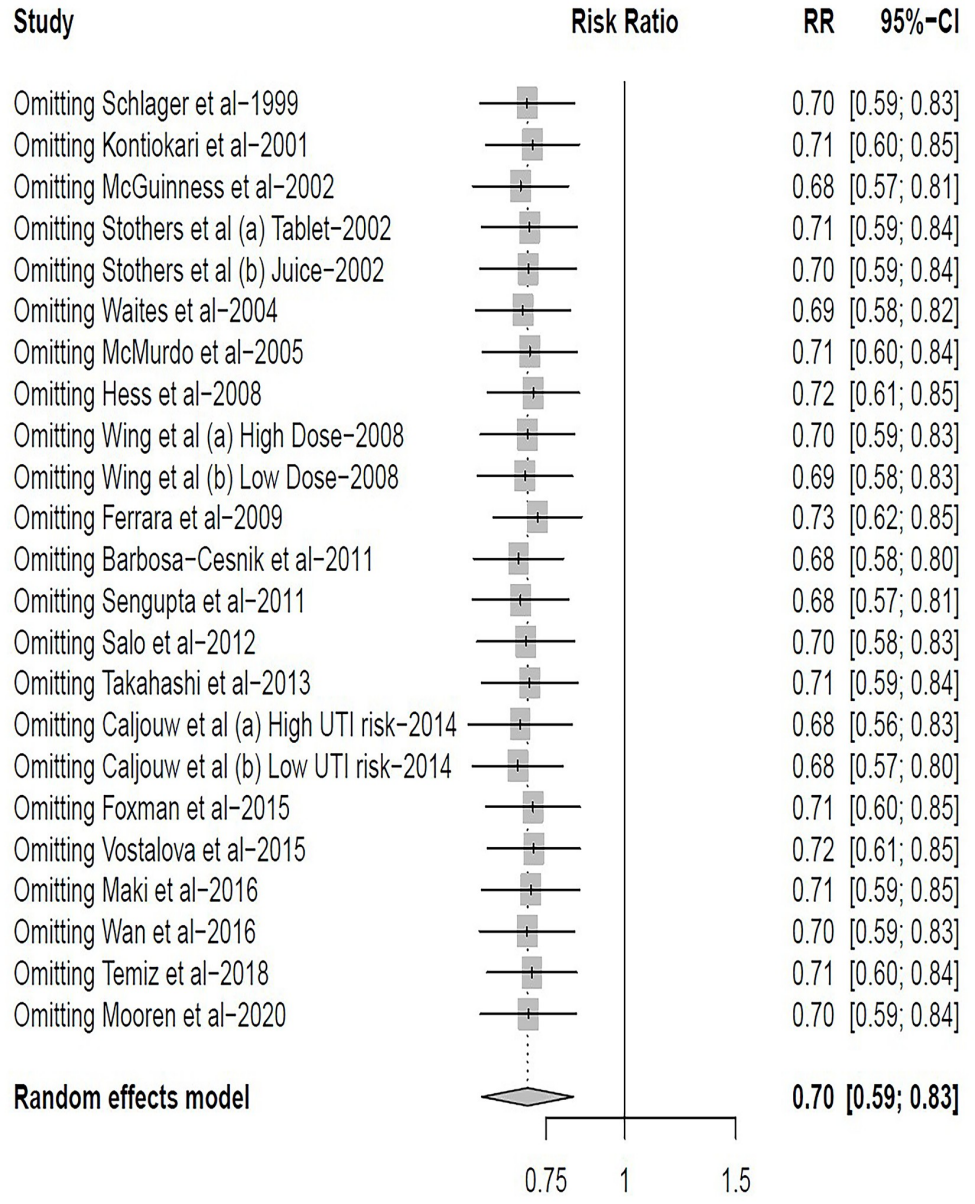
Influential plot: Pooled summary effect estimates with each study omitted at one time.

**Fig 5:**
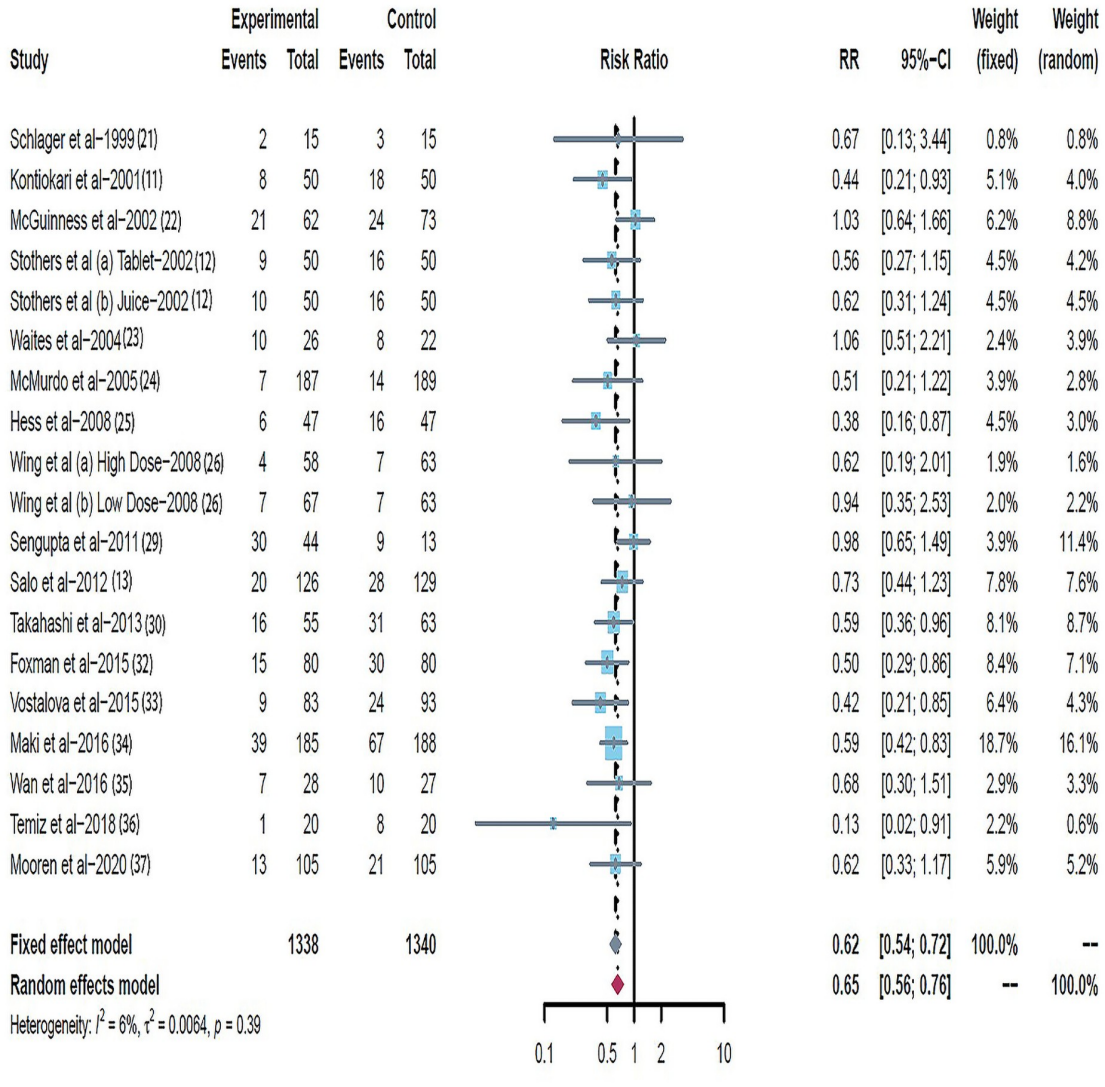
Forest plot: Summary effect of cranberry in prevention of urinary tract infection after excluding three possible sources of heterogeneity, expressed as risk ratio (RR). W(fixed) indicates weights in fixed-effect Mantel-Haenszel model.

Data on UTI cumulative incidence included 3979 participants across the 23 trials [16–35], with 1978 in the cranberry intervention groups and 2001 in the placebo or control groups. There was moderate heterogeneity across trials (RR = 0.70; 95% CI: 0.59 ~ 0.83; I^2^ = 48%) (Fig 2). The Galbraith plot showed that the trials by Ferrara et al. [25], Caljouw et al. [17] and Barbosa-Cesnik et al. [26] were potential sources of heterogeneity (Fig 3). Influential plot further demonstrated that these three trials [17, 25, 26] had significant impact on the pooled summary estimate (Fig 4), justifying its exclusion from the main analysis. After exclusion of these three trials, heterogeneity decreased from 48% to 6% (RR = 0.62; 95% CI: 0.54 ~ 0.79; I^2^ = 6%; P = 0.39) (Fig 5). The results suggested that the trials by Ferrara et al. [25], Caljouw et al. [17] and Barbosa-Cesnik et al. [26] may contribute to the source of heterogeneity.

### Cranberry ingestion effects

**Fig 6:**
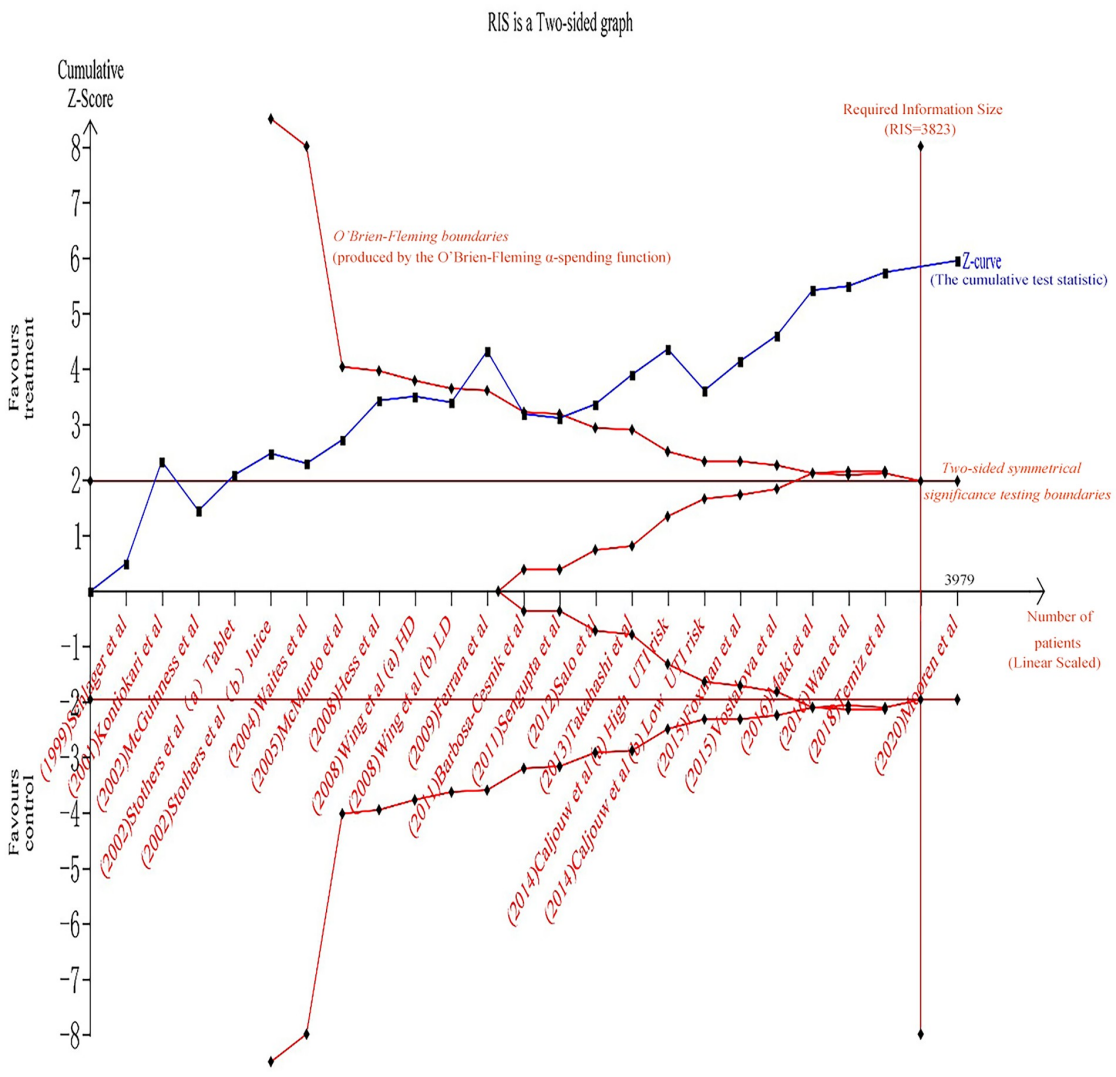
Trial sequential analysis of pooled result of effects of cranberry ingestion on UTI incidence in susceptible populations.

According to all included twenty-three studies, the estimated weighted risk ratio significantly reduced in the risk of recurrent UTIs with cranberry intervention compared to placebo or control (RR = 0.70, 95% CI:0.59 ~ 0.83; P<0.01). There was a moderate degree of heterogeneity (I^2^ = 48%). TSA resulted in a required information size of 3,823, which was reached, and the cumulative Z-curve crossed the boundaries (Fig 6). Therefore, it was possible to reach a conclusion with no need for further additional trials. The application of the TSA of cranberry-containing products ingestion on the incidence of UTIs in susceptible populations was strengthening the conclusions achieved.

### Subgroup and sensitivity analyses

**Fig 7:**
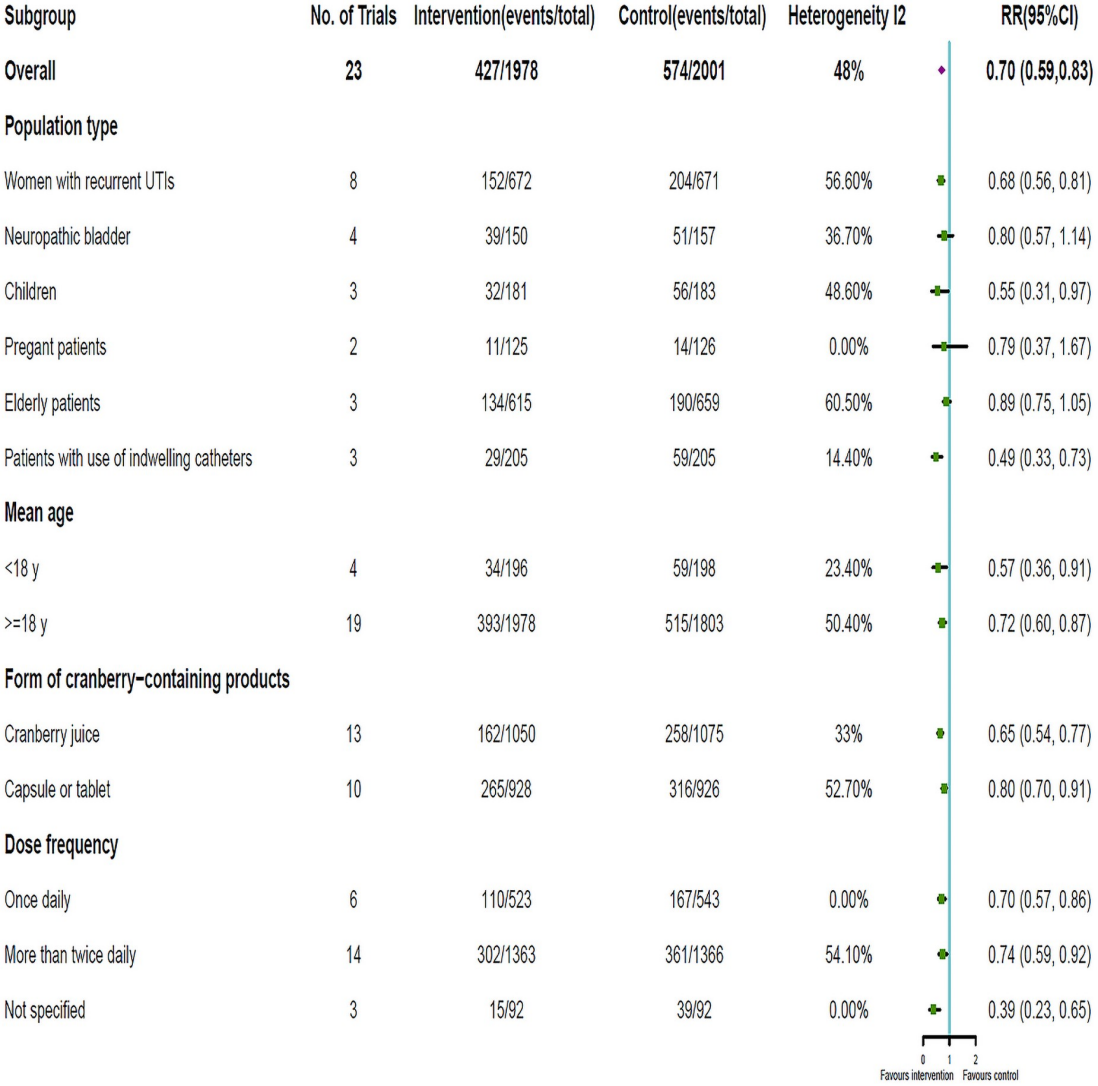
Summary results of subgroup analyses from the included randomized controlled trials evaluating cranberry-containing products in the prevention of UTIs.

A subgroup analysis was conducted to assess the influence of population type, mean patient age, form of cranberry-containing products, and dose frequency on the effectiveness of cranberries in the prevention of UTIs. The effects of cranberry intake on UTIs in subgroup based on characteristics of subjects and interventions are summarized in Fig 7. Although the previous evidence indicated that cranberries may reduce UTIs overall [20, 32], the subgroup analysis showed that compared with placebo or control, cranberries did not significantly decrease recurrent UTIs in some of the subgroups, including patients with neuropathic bladder (RR = 0.80; 95% CI:0.57 ~ 1.14), pregnant patients (RR = 0.79; 95% CI:0.37 ~ 1.67) and elderly patients (RR = 0.89; 95% CI:0.75 ~ 1.05).

Additionally, a sensitivity analysis was performed to estimate the influence of each individual study on pooled RRs. we did not observe a relatively change, which indicated that the summary pooled estimate was stable to study characteristics, treatment in control group and definitions of UTIs (S7 Table). On the whole, the sensitivity analysis revealed that the findings of our meta-analysis were robust.

### Publication bias

**Fig 8:**
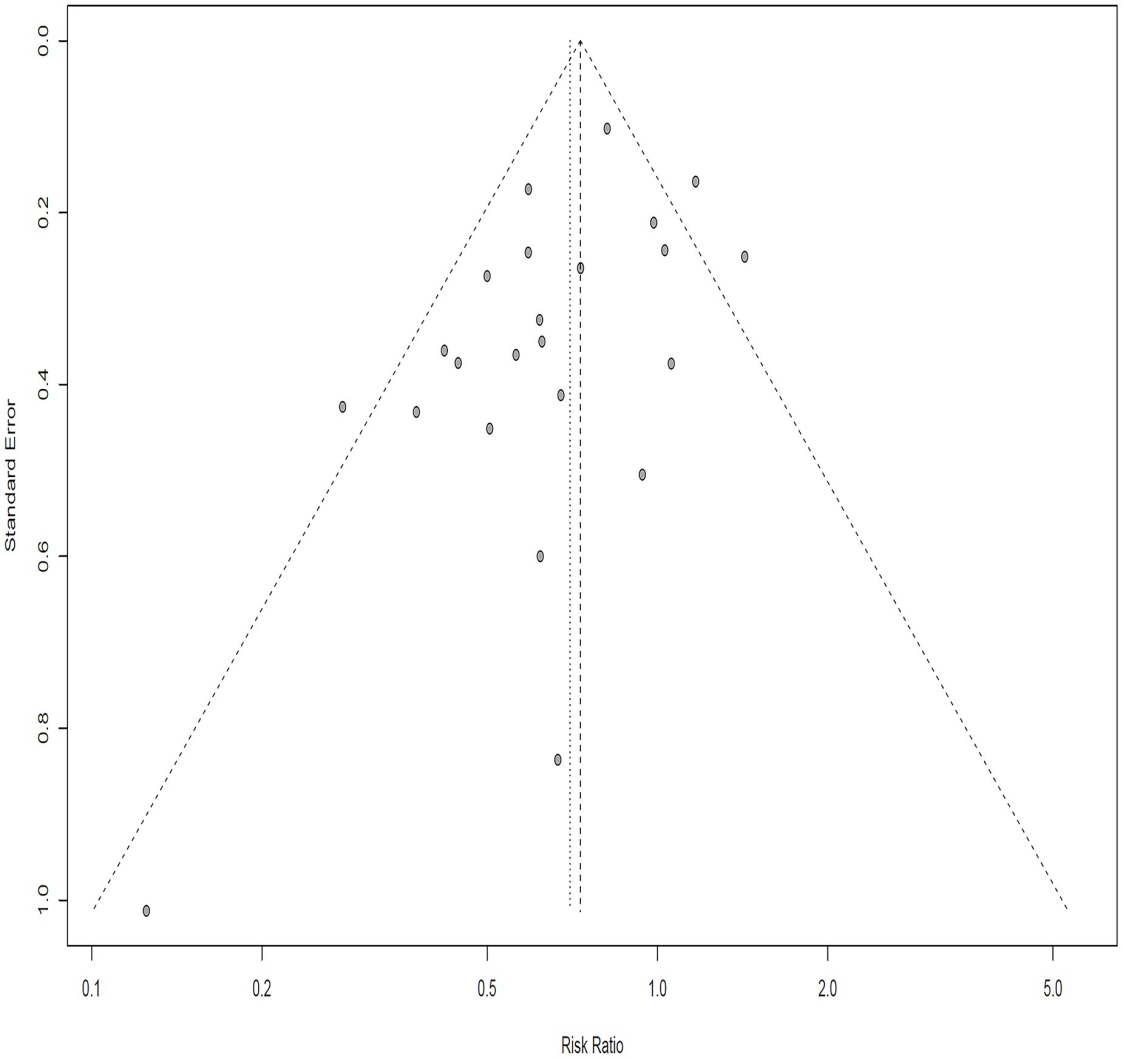
Funnel plot to evaluate publication bias.

In this meta-analysis, A funnel plot was used to evaluate the publication bias qualitatively, and Egger’s regression were used to judge the publication bias quantitatively. Visual scanning of funnel plot suggested no asymmetry with Egger’s regression test (t = -1.34, *p*-value = 0.1956) (Fig 8). Results did not indicate evidence of publication bias.

## Discussion

Our meta-analysis results revealed a 30% reduction in the risk of developing UTI in susceptible populations who consumed cranberry-containing products than those who did not (RR = 0.70; 95% CI: 0.59 ~ 0.83; I^2^ = 48%). We conducted a subgroup analysis to determine the source of heterogeneity. Moreover, we performed meta-regression to explore the potential sources of heterogeneity; however, no statistically significant sources of heterogeneity were identified. The influential and Galbraith plots indicated that three studies were the main sources of heterogeneity [17, 25, 26], and they may explain some unknown heterogeneity.

Barbosa-Cesnik et al. [26] concluded that cranberry juice has no protective effect against UTIs in women compared to those using a placebo(RR = 1.43; 95% CI: 0.87 ~ 2.33). Two potential reasons may account for this study’s deviation. First, unlike previous studies, Barbosa-Cesnik et al. [26] defined UTI using the lowest bacteriuria threshold, which is not less than 1000 colony-forming units [CFU]/mL. According to Kontiokari et al. [20], who used a higher bacteriuria threshold (100,000 CFU/mL), cranberry juice provided positive effects on lowering the recurrence of UTIs. Second, we found that the morbidity of UTIs in the control group in the trial by Barbosa-Cesnik et al. [26] was significantly lower than that in the trial by Kontiokari et al. [20]. Barbosa-Cesnik et al. [26] argued that placebo containing ascorbic acid, might be beneficial to prevent UTI. For the trial by Ferrara et al. [25], they concluded that three treatment arms are among children aged 3–14 years, and children recruited in this study did not meet the original inclusion criteria. In addition, compared to the trial by Salo et al. [28], the intervention amount of cranberry is relatively at a high dose, resulting in a significant difference between the incidence rate of cranberry intervention and control or placebo groups. In the study by Caljouw et al. [17], they evaluated the effectiveness of cranberry capsules among vulnerable elderly. The results indicated that for residents with a high risk of UTI at baseline, taking cranberry capsules twice daily can significantly lower the incidence rate of clinically defined UTI. A possible reason in our analysis for the heterogeneity is that this study used two different criteria for defining UTI, which was distinctly different from that in others studies. When we extracted aggregate data from this study, some errors will be unavoidable. However, it remains unknown whether the population studied in the trial by Ferrara et al. [25], Barbosa-Cesnik et al. [26] and Caljouw et al. [17] were markedly different in other aspects from other trials in our meta-analysis.

Our meta-analysis results exhibit some similarities with previous studies. According to Wang et al. [36] and Jepson et al. [6], consuming cranberry-containing products provided beneficial effects on preventing UTIs. However, Jepson et al. [6] suggested that the benefit of cranberry-containing products might be limited in some subgroups, mostly for women with recurrent UTI, and might be absent in most population groups. We concluded that supplementing cranberry may be beneficial in preventing and treating UTIs in susceptible populations, particularly for women with recurrent UTIs (RR = 0.68; 95% CI: 0.56 ~ 0.81), children (RR = 0.55; 95% CI: 0.31 ~ 0.97) and patients using indwelling catheters (RR = 0.49; 95% CI: 0.33 ~ 0.73).

Our sensitivity analysis revealed that our findings remained stable across study designs, placebo controls, and definitions, and that the effect estimate did not alter significantly when the three studies were excluded (RR = 0.62; 95% CI: 0.54 ~ 0.79; I^2^ = 6%). We conducted a subgroup analysis to explore potential sources of heterogeneity, and discovered no significant sources of heterogeneity between subgroups of age, different individuals, cranberry forms, and dose frequency.

Cranberry in juice form was observed to be more effective than cranberry capsule or tablets in the subgroup analysis. The antibacterial activity of cranberry juice may be due to the intake of large amount of liquid. This result in our analysis might be because people who consuming cranberry juice were better hydrated than those using cranberry capsules or tablets. Additionally, since the precise mechanism of the protective effect of cranberries against UTIs is still not fully elucidated, the better preventive effect of cranberry juice, to some extent, might be due to the additive or synergistic effect of other yet unknown substances in the juice. However, a large volume of cranberry juice with high sugar content may cause severe gastrointestinal symptoms or other adverse effects, as observed by Wing et al. [18], who had to alter their treatment regimens to allow less frequent dosing to maintain compliance and avoid early withdrawal. Notwithstanding, although these adverse effects are a concern, until the exact mechanism behind the protective effects of cranberries against UTIs is clearly comprehended, use of cranberry in juice form might be more favorable than cranberry in capsules or tablets.

The present systematic review and meta-analysis with TSA indicate that cranberry intake can prevent UTIs in susceptible populations. However, although European Association of Urology recommends regular consumption of cranberries as a nutritive method to effectively prevent UTIs, the data from relevant studies are inconclusive because few available studies lasted longer than a year. As a result, there is insufficient evidence to support the efficacy of cranberry products in clinical use. Future studies should continue to be performed for a longer period.

The treatment effect of cranberry products may depend on their PAC concentration. Currently, the most recognized mechanism by which cranberry can prevent UTIs usually involves its interference with bacterial adhesion in the urinary tract [37]. Cranberry consumption can cause an antiadhesion response in urine. If bacteria are unable to adhere to cells, they cannot grow and cause infection [37].

This meta-analysis possessed several strengths. Our study examined the efficacy of cranberry-containing products in susceptible populations and used trial sequential analysis to determine whether the evidence for cranberry consumption preventing UTIs in susceptible populations is sufficient. Additionally, it was performed and reported based on current guidelines [38, 39], and comprised an evaluation of results employing numerous sensitivity analyses, as well as investigation of the risk of bias using an updated assessment tool. In addition, there were potential limitations to our review.: first, insufficient information in several included studies caused certain limitations. We attempted to contact the authors three times but were unable to obtain any pertinent information. Second, one study used a mixture of juice concentrate including cranberry juice and lingonberry juice [20]. Lingonberry (Vaccinium *visit-idaea*) and Cranberry would most likely contain similar phytochemical composition, so the observed beneficial effects may not be entirely attributed to cranberry alone. Third, there was lack of in-consistency among the doses of PACs used in the included studies. The daily recommended intake of PACs, to decrease the number of recurrent UTIs, is not lower than 36 mg, and inconsistent dosages between different studies may cause different outcomes.

## Conclusion

In summary, evidence from our updated meta-analysis indicated that cranberry supplementation significantly reduced the incidence of occurring UTIs in susceptible populations. Furthermore, cranberry may be considered as a promising adjuvant therapy for preventing UTIs in susceptible individuals. However, due to some limits of the included trials in this review, the conclusion therefore should be interpreted with caution. Further high-quality studies with appropriate large sample size are required to verify our results.

## Supporting information

S1 Table Detailed search strategy. (DOCX)

S2 Table Study design of the 28 included randomized controlled trials evaluating cranberry-containing products in the prevention of UTI. (PDF)

S3 Table Quality of reporting of the 28 included randomized controlled trials evaluating cranberry-containing products in the prevention of UTI. (PDF)

S4 Table Characteristics of study populations in the 28 included randomized controlled trials evaluating cranberry-containing products in the prevention of UTIs. (PDF)

S5 Table Characteristics of interventions in the 28 included randomized controlled trials evaluating cranberry-containing products in the prevention of UTIs. (PDF)

S6 Table Definitions and outcomes from the 28 included randomized controlled trials evaluating cranberry-containing products in the prevention of UTIs. (PDF)

S7 Table Summary results of sensitivity analyses from the included randomized controlled trials evaluating cranberry-containing products in the prevention of UTIs. (PDF)

S1 Checklist PRISMA 2009 checklist. (DOC)
