# Supplementary material for: Consumption of cranberry as adjuvant therapy for urinary tract infections in susceptible populations: A systematic review and meta-analysis with trial sequential analysis
Source: PLoS One. 2021 Sep 2;16(9):e0256992. doi: 10.1371/journal.pone.0256992 (PMC8412316; doi:10.1371/journal.pone.0256992)
Supplement: S1 Table — (DOCX) [file pone.0256992.s001.docx]

| #1 Cranberry |
| --- |
| #2 Vaccinium macrocarpon |
| #3 Vaccinium microcarpum |
| #4 Vaccinium oxycoccus |
| #5 Vaccinium erythrocarpum |
| #6 Urinary Tract Infection OR Urinary Tract Infections |
| #7 UTI |
| #8 bacteriuria |
| #9 pyelonephritis |
| #10 cystitis |
| #11 pyuria |
| #12 dysuria |
| #13 Escherichia coli or coli |
| #14 1 or 2 or 3 or 4 or 5 |
| #15 6 or 7 or 8 or 9 or 10 or 11 or 12 or 13 |
| #16 #14 AND #15 |

**S1 Table. Detailed search strategy**

**PubMed search 2021.06.30**

**All individual searches from each database inception date until 30 June 2021**

**Embase search 2021.06.30**

| #1 Cranberry |
| --- |
| #2 Vaccinium macrocarpon |
| #3 Vaccinium microcarpum |
| #4 Vaccinium oxycoccus |
| #5 Vaccinium erythrocarpum |
| #6 Urinary Tract Infection* OR UTI* |
| #7 bacteriuria |
| #8 pyelonephritis |
| #9 cystitis |
| #10 pyuria |
| #11 dysuria |
| #12 Escherichia coli OR coli |
| #13 1 or 2 or 3 or 4 or 5 |
| #14 6 or 7 or 8 or 9 or 10 or 11 or 12 |
| #15 #13 AND #14 |

**All individual searches from each database inception date until 30 June 2021**

**Cochrane search 2021.06.30**

| #1 Cranberry |
| --- |
| #2 Vaccinium macrocarpon OR Vaccinium microcarpum OR Vaccinium oxycoccus OR Vaccinium erythrocarpum |
| #3 1 or 2 |
| #4 Urinary Tract Infection* OR UTI* |
| #5 bacteriuria |
| #6 pyelonephritis |
| #7 cystitis |
| #8 pyuria |
| #9 dysuria |
| #10 Escherichia coli OR coli |
| #11 4 or 5 or 6 or 7 or 8 or 9 or 10 |
| #12 #3 AND #11 |

**All individual searches from each database inception date until 30 June 2021**

**Web of science search 2021.06.30**

| #1 Cranberry |
| --- |
| #2 Vaccinium macrocarpon OR Vaccinium microcarpum OR Vaccinium oxycoccus OR Vaccinium erythrocarpum |
| #3 1 or 2 |
| #4 Urinary Tract Infection* OR UTI* |
| #5 bacteriuria |
| #6 pyelonephritis |
| #7 cystitis |
| #8 pyuria |
| #9 dysuria |
| #10 Escherichia coli OR coli |
| #11 4 or 5 or 6 or 7 or 8 or 9 or 10 |
| #12 #3 AND #11 |

**All individual searches from each database inception date until 30 June 2021**

**Scopus search 2021.06.30**

| #1 Cranberry |
| --- |
| #2 Vaccinium macrocarpon |
| #3 Vaccinium microcarpum |
| #4 Vaccinium oxycoccus |
| #5 Vaccinium erythrocarpum |
| #6 Urinary Tract Infection* OR UTI* |
| #7 bacteriuria |
| #8 pyelonephritis |
| #9 cystitis |
| #10 pyuria |
| #11 dysuria |
| #12 Escherichia coli OR coli |
| #13 1 or 2 or 3 or 4 or 5 |
| #14 6 or 7 or 8 or 9 or 10 or 11 or 12 |
| #15 #13 AND #14 |

**All individual searches from each database inception date until 30 June 2021**
