# Supplementary material for: Consumption of cranberry as adjuvant therapy for urinary tract infections in susceptible populations: A systematic review and meta-analysis with trial sequential analysis
Source: PLoS One. 2021 Sep 2;16(9):e0256992. doi: 10.1371/journal.pone.0256992 (PMC8412316; doi:10.1371/journal.pone.0256992)
Supplement: S2 Table — (PDF) [file pone.0256992.s002.pdf]

S1 Table. Study Design of the 28 Included Randomized Controlled Trials Evaluating Cranberry-Containing Products in the Prevention of UTI

Source	Design	Washout period	Analysis	Study Duration	Loss to Follow-up, No./Total No. (%)
Avorn et al,1994	Parallel	NA	ITT	6 mo	32/153 (21)
Foda et al,1995	Crossover	None	PP	12mo	19/40 (48)
Walker et al,1997	Crossover	None	PP	6 mo	9/19 (47)
Schlager et al,1999	Crossover	None	ITT	6 mo	0/15 (0)
Kontiokari et al,2001	Parallel	NA	ITT	6 mo	9/50 (18)
McGuinness et al,2002	Parallel	NA	ITT	6 mo	12/135 (9)
Stothers et al (a) Tablet-2002	Parallel	NA	ITT	12mo	NR
Stothers et al (b) Juice-2002	Parallel	NA	ITT	12mo	NR
Waites et al,2004	Parallel	NA	PP	6 mo	26/74 (35)

McMurdo et al,2005	Parallel	NA	ITT	35 d	115/376 (30.6)
Hess et al,2008	Crossover	None	PP	12mo	10/57 (18)
Wing et al (a) High Dose -2008	Parallel	NA	ITT	6 mo [a]	73/188 (39)
Wing et al (b) Low Dose-2008	Parallel	NA	ITT	6 mo [a]	73/188 (39)
Ferrara et al,2009	Parallel	NA	PP	6 mo	3/57 (5)
Barbosa-Cesnik et al,2011	Parallel	NA	ITT	6 mo	89/319 (27.9)
Sengupta et al,2011	Parallel	NA	PP	90 d	3/60 (5)
Stapleton et al,2012	Parallel	NA	ITT	6 mo	66/186 (35)
Salo et al,2012	Parallel	NA	PP	6 mo	8/263 (3)
Afshar et al,2012	Parallel	NA	ITT	12mo	12/40 (30)
Takahashi et al,2013	Parallel	NA	PP	24 w	24/237 (10)
Caljouw et al (a) High UTI risk-2014	Parallel	NA	ITT	12mo	331/928 (36)
Caljouw et al (b) Low UTI risk-2014	Parallel	NA	ITT	12mo	331/928 (36)

Foxman et al,2015	Parallel	NA	ITT	6 w	7/180 (4)
Vostalova et al,2015	Parallel	NA	ITT	6 mo	17/182 (9)
Ledda et al,2015	Parallel	NA	ITT	60 d	NR
Juthani-Mehta et al,2016	Parallel	NA	ITT	12mo	38/185 (21)
Maki et al,2016	Parallel	NA	ITT	24 w	21/373 (6)
Wan et al,2016	Parallel	NA	ITT	6 mo	0
Ostrovsky et al,2017	Parallel	NA	ITT	360 d	NR (20)
Temiz et al,2018	Parallel	NA	PP	3 mo	27/87 (31)
Mooren et al,2020	Parallel	NA	ITT	6 w	0

ITT=Intention To Treat, NA=Not Applicable, NR=Not Reported, PP=Per Protocol. [a] Pregnant women were followed from prior to week 16 of gestation through delivery.
