# Supplementary material for: Consumption of cranberry as adjuvant therapy for urinary tract infections in susceptible populations: A systematic review and meta-analysis with trial sequential analysis
Source: PLoS One. 2021 Sep 2;16(9):e0256992. doi: 10.1371/journal.pone.0256992 (PMC8412316; doi:10.1371/journal.pone.0256992)
Supplement: S3 Table — (PDF) [file pone.0256992.s003.pdf]

S2 Table. Quality of Reporting of the 28 Included Randomized Controlled Trials Evaluating Cranberry-Containing Products in the Prevention of UTI

Reference	Risk of Bias Assessment						
	Random Sequence Generation	Allocation Concealment	Blinding of participants and personnel	Blinding of outcome assessment	Incomplete outcome data	Selective Reporting	Other bias
Avorn et al,1994	H	H	L	L	H	H	U
Foda et al,1995	U	U	L	L	H	H	U
Walker et al,1997	U	U	L	L	H	U	U
Schlager et al,1999	U	U	L	L	L	H	U
Kontiokari et al,2001	L	L	L	L	H	L	U
McGuinness et al,2002	U	U	L	L	H	H	U
Stothers et al (a) Tablet-2002	L	L	H	U	L	U	U

Stothers et al (b) Juice-2002	L	L	H	U	L	U	U
Waites et al,2004	U	U	L	L	H	H	L
McMurdo et al,2005	L	L	L	L	L	H	L
Hess et al,2008	U	U	L	L	U	H	L
Wing et al (a) High Dose-2008	L	U	L	L	H	H	U
Wing et al (b) Low Dose-2008	L	U	L	L	H	H	U
Afshar et al,2012	L	H	L	L	U	L	U
Takahashi et al,2013	U	L	L	L	L	L	L
Caljouw et al (a) High UTI risk-2014	U	L	L	L	L	L	U
Caljouw et al (b) Low UTI risk-2014	U	L	L	L	L	L	U
Foxman et al,2015	L	L	L	L	L	L	L
Vostalova et al,2015	U	L	L	U	L	H	H
Ledda et al,2015	U	U	U	U	L	U	U

Juthani-Mehta et al,2016	L	L	L	L	L	L	L
Maki et al,2016	L	U	L	L	L	L	L
Wan et al,2016	L	L	L	L	L	L	U
Ostrovsky et al,2017	L	U	U	U	L	U	U
Temiz et al,2018	L	U	L	L	L	L	U
Juthani-Mehta et al,2016	L	L	L	L	L	L	L
Maki et al,2016	L	U	L	L	L	L	L
Wan et al,2016	L	L	L	L	L	L	U
Ostrovsky et al,2017	L	U	U	U	L	U	U
Temiz et al,2018	L	U	L	L	L	L	U
Mooren et al,2020	L	L	L	L	L	L	L

U=unclear risk of bias, L=low risk of bias, H=high risk of bias.
