# Supplementary material for: Consumption of cranberry as adjuvant therapy for urinary tract infections in susceptible populations: A systematic review and meta-analysis with trial sequential analysis
Source: PLoS One. 2021 Sep 2;16(9):e0256992. doi: 10.1371/journal.pone.0256992 (PMC8412316; doi:10.1371/journal.pone.0256992)
Supplement: S4 Table — (PDF) [file pone.0256992.s004.pdf]

S3 Table. Characteristics of Study Populations in the 28 Included Randomized Controlled Trials Evaluating Cranberry-Containing Products in the Prevention of UTIs

Source	No. of patients	Study region	Setting	Age, Range (Mean or Median), y	Subgroup	Female, %
Avorn et al,1994	153	US	Nursing homes	NA (78.6)	Elderly patients	100
Foda et al,1995	21	Canada	Hospital clinic	1.4-18 (NA)	Patients with neuropathic bladder	43
Walker et al,1997	10	US	Unclear	28-44 (37)	Women with recurrent UTIs	100
Schlager et al,1999	15	US	Hospital clinic	2-18 (NA)	Patients with neuropathic bladder	53
Kontiokari et al,2001	100	Finland	Hospital clinic	NA (30.5)	Women with recurrent UTIs	100
McGuinness et al,2002	135	Canada	Hospital clinic	NA (45.1)	Patients with neuropathic bladder	79
Stothers et al (a) Tablet-2002	150	Canada	Unclear	21-72 (42.3)	Women with recurrent UTIs	100

Stothers et al (b) Juice-2002	150	Canada	Unclear	21-72 (42.3)	Women with recurrent UTIs	100
Waites et al,2004	48	US	Hospital clinic	20-73 (40.9)	Patients with neuropathic bladder	13
McMurdo et al,2005	376	UK	Inpatients	＞60 (81.4)	Elderly patients	68
Hess et al,2008	47	US	Hospital clinic	28-79 (53)	Patients with neuropathic bladder	0
Wing et al (a) High Dose-2008	188	US	Hospital clinic	NA (26.4)	Pregnant women	100
Wing et al (b) Low Dose-2008	188	US	Hospital clinic	NA (26.4)	Pregnant women	100
Ferrara et al,2009	54	Italy	Hospital clinic	3-14 (NA)	Children	100
Barbosa-Cesnik et al,2011	319	US	Home	18-40 (21.2)	Women with recurrent UTIs	100
Sengupta et al,2011	60	India	Hospital clinic	18-40 (31.7)	Women with recurrent UTIs	100

Stapleton et al,2012	176	US	Hospital clinic	18-45 (25)	Premenopausal women with a history of recent UTI	100
Salo et al,2012	263	Finland	Hospital clinic	1-16 (NA)	Children with verified UTI	91
Afshar et al,2012	40	Canada	Hospital clinic	＜=18 (7)	Children with documented symptomatic UTIs	98
Takahashi et al,2013	150	Japan	Urology clinic	20-79 (NA)	Patients with cystitis	NA
Caljouw et al (a) High UTI risk-2014	928	Holland	Long-term care facilities	>=65 (84)	Long-term care facilities residents	76
Caljouw et al (b) Low UTI risk-2014	928	Holland	Long-term care facilities	>=65 (84)	Long-term care facilities residents	76
Foxman et al,2015	160	US	Hospital clinic	>18 (NA)	Women undergoing gynecological surgery and indwelling catheter	100
Vostalova et al,2015	182	Czech Republic	Hospital clinic	18-75 (36.7)	Women with recurrent UTIs	100

Ledda et al,2015	44	Italy	Unclear	NA (39)	Patients with a history of recurrent UTI	75
Juthani-Mehta et al,2016	185	US	Nursing Homes	>=65 (86.4)	Older Women	100
Maki et al,2016	373	US	Hospital clinic	20-70 (40.9)	Women with recurrent UTIs	100
Wan et al,2016	67	Taiwan, China	Hospital clinic	6-18 (9.5)	Uncircumcised Boys	0
Ostrovsky et al,2017	185	US	Nursing Homes	NA (86)	Women	100
Temiz et al,2018	60	Turkey	Hospital clinic	>=18 (63.8)	Patients with ileal conduit diversion	32
Mooren et al,2020	210	The Netherlands	Hospital clinic	>18 (62.5)	Women with pelvic floor surgery and indwelling catheter	100

PAC=A-type proanthocyanidin, UK=United Kingdom, US=United States, UTIs=urinary tract infections.
