# Supplementary material for: Consumption of cranberry as adjuvant therapy for urinary tract infections in susceptible populations: A systematic review and meta-analysis with trial sequential analysis
Source: PLoS One. 2021 Sep 2;16(9):e0256992. doi: 10.1371/journal.pone.0256992 (PMC8412316; doi:10.1371/journal.pone.0256992)
Supplement: S5 Table — (PDF) [file pone.0256992.s005.pdf]

S4 Table. Characteristics of Interventions in the 28 Included Randomized Controlled Trials Evaluating Cranberry-Containing Products in the Prevention of UTIs

		Cranberry Group				Control Group
**Source**	**Baseline Bacteriuria Excluded?**	**Form (Daily Dosage, mL)**	**Manufacturer**	**Cranberry Amount (g/d) (PAC Content, mg/d)**	**Dosing Frequency (Daily)**	**Formula**
Avorn et al,1994	No	Juice (300)	Ocean Spray	NA	Not specified	Juice [a]
Foda et al,1995	No	Juice (15 mL/kg)	Ocean Spray	4.5 g/kg/d	3-4	Water
Walker et al,1997	No	Capsule	Solaray	0.4	Not specified	Dicalcium Phosphate
Schlager et al,1999	No	Juice (60)	Ocean Spray	NA	Not specified	Juice [a]

Kontiokari et al,2001	Yes	Juice (50)	Marli	7.5	Not specified	No placebo
McGuinness et al,2002	No	Capsule	NOW Natural Foods	8	1	Beetroot
Stothers et al (a) Tablet-2002	Yes	Tablet (750)	Unclear	NA	2/3	Juice [b]
Stothers et al (b) Juice-2002	Yes	Juice (750)	Unclear	NA	2/3	Juice [b]
Waites et al,2004	No	Capsule	Aim This Way	4	2	Lactose
McMurdo et al,2005	No	Juice (300)	Ocean Spray	75 (0.838)	2	Juice [c]
Hess et al,2008	Yes	Tablet	Swiss Herbal	1	2	Rice flour
Wing et al (a) High Dose -2008	Yes	Juice (720)	Ocean Spray	194.4 (318)	3	Juice [a]
Wing et al (b) Low Dose-2008	Yes	Juice (240)	Ocean Spray	64.8(106)	1	Juice [a]
Ferrara et al,2009	Yes	Juice (50)	Unclear	7.5	Not specified	No placebo
Barbosa-Cesnik et al,2011	Yes	Juice (480)	Ocean Spray	129.6 (224)	2	Juice [a]

Sengupta et al,2011	No	Capsule	Decas Botanical Synergies	0.1 or 1g/d	2	No placebo
Stapleton et al,2012	Yes	Juice (473 or 946)	Ocean Spray	NA	1	Juice [a]
Salo et al,2012	No	Juice (5 mL/kg)	Ocean Spray	0.21g/kg/d	1-2	Juice [a]
Afshar et al,2012	No	Juice (2 mL/kg)	Ocean Spray	NA	1	Placebo juice
Takahashi et al,2013	No	Juice (125)	The Nisshin Oillio Group	20-79 (NA)	1	Placebo beverage
Caljouw et al (a) High UTI risk-2014	No	Capsule	Unclear	>=65 (84)	2	Placebo capsule
Caljouw et al (b) Low UTI risk-2014	No	Capsule	Unclear	>=65 (84)	2	Placebo capsule
Foxman et al,2015	Yes	Capsule	Theralogix, LLC	Unclear	4	Placebo capsule

Vostalova et al,2015	Yes	Capsule	NA TUREX-DBS	0.5g/d (2.8mg)	2	Placebo capsule
Ledda et al,2015	No	Capsule	Unclear	Unclear	1	No placebo
Juthani-Mehta et al,2016	Yes	Capsule	Pharmatoka	0.072g/d	2	Placebo capsule
Maki et al,2016	No	Juice (240)	Ocean Spray	NA	1	Juice [a]
Wan et al,2016	No	Juice (120)	Ocean Spray	NA	1	Placebo juice
Ostrovsky et al,2017	No	Capsule (590)	Unclear	Unclear	2	Placebo capsule
Temiz et al,2018	No	Capsule	Unclear	0.8(18)	2	No placebo
Mooren et al,2020	No	Capsule	OrthoBasics, Midwoud	NA (72)	2	Placebo capsule

[a] Specially designed juice to imitate cranberry juice.

[b] Filtered water with food coloring plus 20-mL pineapple juice. [c] Containing water, sucrose, elderberry extract, quinic acid, citric acid, malic acid, vitamin C, and aspartame.
