# Supplementary material for: Consumption of cranberry as adjuvant therapy for urinary tract infections in susceptible populations: A systematic review and meta-analysis with trial sequential analysis
Source: PLoS One. 2021 Sep 2;16(9):e0256992. doi: 10.1371/journal.pone.0256992 (PMC8412316; doi:10.1371/journal.pone.0256992)
Supplement: S6 Table — (PDF) [file pone.0256992.s006.pdf]

S5 Table. Definitions and Outcomes From the 28 Included Randomized Controlled Trials Evaluating Cranberry-Containing Products in the Prevention of UTIs

	Outcome Assessment			UTI Cumulative Incidence Rate, No./No. (%)		UTI Incidence Rate (Episodes/ Patient-year)		
Source	Threshold of Bacteriuria (CFU/mL)	Pyuria Required in Definition of UTI	Symptoms Required in Definition of UTI	Cranberry	Control	Cranberry	Control	Intervention Duration [a]
Avorn et al,1994	100 000	Yes (undefined)	No	0.15% [b]	28.1% [b]	NA	NA	6 mo
Foda et al,1995	100 000	No	Yes (undefined)	NA	NA	1.8	1.9	6 mo
Walker et al,1997	Undefined	No	Yes (undefined)	NA	NA	2.4	6.0	3 mo

Schlager et al,1999	10 000	No	Yes (defined)	2/15 (13)	3/15 (20)	0.8	0.8	3 mo
Kontiokari et al,2001	100 000	No	Yes (defined)	8/50 (16)	18/50 (36)	NA	NA	6 mo
McGuinness et al,2002	1 000 000	Yes (undefined)	No	21/62 (34)	24/73 (33)	NA	NA	6 mo
Stothers et al (a) Tablet-2002	100 000	No	Yes (undefined)	9/50 (18)	16/50 (32)	0.4	0.7	12 mo
Stothers et al (b) Juice-2002	100 000	No	Yes (undefined)	10/50(20)	16/50 (32)	0.3	0.7	12 mo
Waites et al,2004	10 000	No	Yes (defined)	10/26 (34)	8/22 (36)	1.2	1.3	6 mo
McMurdo et al,2005	10 000	No	Yes (defined)	7/187 (4)	14/189 (7)	NA	NA	35 d
Hess et al,2008	10 000	Yes (defined)	Yes (defined)	6/47 (13)	16/47 (34)	0.3	0.9	6 mo
Wing et al (a) High Dose-2008	100 000	Yes (defined)	Yes (defined)	4/58(7)	7/63 (11)	NA	NA	6 mo
Wing et al (b) Low Dose-2008	100 000	Yes (defined)	Yes (defined)	7/67 (10)	7/63 (11)	NA	NA	6 mo

Ferrara et al,2009[1]	100 000	Yes (defined)	Yes (defined)	5/27 (19)	18/27 (67)	NA	NA	6 mo
Barbosa-Cesnik et al,2011	1000	Yes (defined)	Yes (defined)	31/155 (20)	23/164 (14)	NA	NA	6 mo
Sengupta et al,2011	Undefined	No	No	30/44(68)	9/13(69)	NA	NA	90 D
Stapleton et al,2012	100 000	Yes (defined)	Yes (defined)	0.29	0.37	NA	NA	6 mo
Salo et al,2012	100 000	No	Yes (defined)	20/126(16)	28/129(22)	0.25	0.41	6 mo
Afshar et al,2012	100 000	No	Yes (defined)	NA	NA	0.4	1.15	12 mo
Takahashi et al,2013	Undefined	No	No	16/55(29)	31/63(49)	NA	NA	24w
Caljouw et al (a) High UTI risk-2014	Undefined	No	No	98/253 (39)	125/263 (48)	62.8	84.8	12mo
Caljouw et al (b) Low UTI risk-2014	Undefined	No	No	59/205 (29)	51/207(25)	NA	NA	12mo
Foxman et al,2015	Undefined	No	No	15/80(19)	30/80(38)	NA	NA	6w

Vostalova et al,2015	100 000	No	Yes (defined)	9/83 （11）	24/93 （26）	NA	NA	6 mo
Ledda et al,2015	Undefined	No	No	NA	NA	NA	NA	60 D
Juthani-Mehta et al,2016	100 000	Yes (defined)	Yes (defined)	25.5%	29.5%	NA	NA	12 mo
Maki et al,2016	100 000	No	Yes (defined)	39/185(21)	67/188(36)	NA	NA	24w
Wan et al,2016	100 000	No	No	7/28(25)	10/27(37)	NA	NA	6 mo
Ostrovsky et al,2017	100 000	No	No	29.1	29	NA	NA	360 D
Temiz et al,2018	100 000	No	No	1/20(5)	8/20(40)	NA	NA	3 mo
Mooren et al,2020	Undefined	No	No	13/105(12)	21/105(20)	NA	NA	6w

CFU=colony-forming unit, NA= Not Available, UTIs=Urinary Tract Infections.

[a] Intervention duration: In crossover trials, intervention duration of each intervention was half of the study duration.

[b] This percentage represented urine samples of bacteriuria with pyuria in all screened urine samples. The odds ratio was 0.42 (95% CI, 0.23-0.76) for bacteriuria with pyuria in cranberry relative to the control group (*P* = .004).
