# Supplementary material for: Consumption of cranberry as adjuvant therapy for urinary tract infections in susceptible populations: A systematic review and meta-analysis with trial sequential analysis
Source: PLoS One. 2021 Sep 2;16(9):e0256992. doi: 10.1371/journal.pone.0256992 (PMC8412316; doi:10.1371/journal.pone.0256992)
Supplement: S7 Table — (PDF) [file pone.0256992.s007.pdf]

S6 Table. Summary Results of Sensitivity Analyses From the Included Randomized Controlled Trials Evaluating Cranberry-Containing Products in the Prevention of UTIs

Sensitivity Analysis According to:	No. of Trials	Pooled Cumulative Incidence Rate of UTI, No./No. [a]		Heterogeneity I^2, % (*P* Value)	Risk Ratio (95% CI)	*P* Value in Meta-regression
		Cranberry	Control			
**Study design**						
Parallel	21	419/1916	555/1939	49.2 (0.006)	0.74 (0.67, 0.83)	0.265
Crossover	2	39/150	51/157	0 (0.541)	0.42 (0.20, 0.89)	
**Study analysis**						
ITT	16	339/1633	456/1680	44.2 (0.030)	0.77 (0.68, 0.86)	0.478
PP	7	143/400	118/321	60.8 (0.018)	0.60(0.47, 0.76)	
**Treatment in control group**						
Placebo	19	383/1837	521/1869	38.9 (0.043)	0.76(0.68, 0.85)	0.262

No placebo	4	44/141	53/110	80.1 (0.002)	0.48 (0.33, 0.68)	
**Bacteriuria threshold in definition of UTI**						
10 000 CFU/mL	4	25/275	41/273	18.7 (0.297)	0.58 (0.37, 0.91)	0.681
100 000 CFU/mL	11	119/726	219/760	0 (0.584)	0.55 (0.45, 0.67)	
**Pyuria requirement in definition of UTI**						
Pyuria unrequired	17	346/1537	479/1564	37.9 (0.058)	0.71 (0.63, 0.80)	0.557
Pyuria required	6	74/416	95/437	68.2 (0.008)	0.83(0.63, 1.08)	

ITT=Intention To Treat, PP=Per Protocol, UTIs=Urinary Tract Infections.

[a] Pooled incidence rate: number of patients who experienced at least 1 episode of UTI/number of patients at risk during the intervention period.
